# Pre-Columbian Origins for North American Anthrax

**DOI:** 10.1371/journal.pone.0004813

**Published:** 2009-03-13

**Authors:** Leo J. Kenefic, Talima Pearson, Richard T. Okinaka, James M. Schupp, David M. Wagner, Jacques Ravel, Alex R. Hoffmaster, Carla P. Trim, Wai-Kwan Chung, Jodi A. Beaudry, Jeffrey T. Foster, James I. Mead, Paul Keim

**Affiliations:** 1 Department of Biological Sciences, Northern Arizona University, Flagstaff, Arizona, United States of America; 2 The Institute for Genome Sciences, University of Maryland School of Medicine, Baltimore, Maryland, United States of America; 3 Bacterial Zoonoses Branch, Centers for Disease Control and Prevention, Atlanta, Georgia, United States of America; 4 Pathogen Genomics Division, Translational Genomics Research Institute, Phoenix, Arizona, United States of America; St. Petersburg Pasteur Institute, Russian Federation

## Abstract

Disease introduction into the New World during colonial expansion is well documented and had a major impact on indigenous populations; however, few diseases have been associated with early human migrations into North America. During the late Pleistocene epoch, Asia and North America were joined by the Beringian Steppe ecosystem which allowed animals and humans to freely cross what would become a water barrier in the Holocene. Anthrax has clearly been shown to be dispersed by human commerce and trade in animal products contaminated with *Bacillus anthracis* spores. Humans appear to have brought *B. anthracis* to this area from Asia and then moved it further south as an ice-free corridor opened in central Canada ∼13,000 ybp. In this study, we have defined the evolutionary history of Western North American (WNA) anthrax using 2,850 single nucleotide polymorphisms (SNPs) and 285 geographically diverse *B. anthracis* isolates. Phylogeography of the major WNA *B. anthracis* clone reveals ancestral populations in northern Canada with progressively derived populations to the south; the most recent ancestor of this clonal lineage is in Eurasia. Our phylogeographic patterns are consistent with *B. anthracis* arriving with humans via the Bering Land Bridge. This northern-origin hypothesis is highly consistent with our phylogeographic patterns and rates of SNP accumulation observed in current day *B. anthracis* isolates. Continent-wide dispersal of WNA *B. anthracis* likely required movement by later European colonizers, but the continent's first inhabitants may have seeded the initial North American populations.

## Introduction

The basic premises of disease tracking have changed little since John Snow first described the London cholera epidemic of 1854. The use of molecular genotyping technologies has allowed the epidemiological linkage of geographically disparate isolates, generating hypotheses about patterns and modes of disease dispersal. As might be expected, the distribution of human pathogens that cause persistent infections such as *Helicobacter pylori*, the Typhi serovar of *Salmonella enterica*, *Mycobacterium tuberculosis* and Polyomavirus JC reflect both recent and ancient human migratory patterns [1]–[7]. Conversely, pathogens that cause acute infections remain only briefly within a host and are therefore less likely to follow long term host distribution patterns [3]. The dispersal of *Bacillus anthracis*, *Yersinia pestis*, and human RNA viruses often reflect short term human movement frequently associated with trading contaminated animal products or inadvertently transporting primary vectors or hosts [1], [3], [8]–[12]. Such potentially frequent and long range dispersal of pathogens can obscure more ancient phylogeographic patterns. The history of *B. anthracis* in North America has certainly been affected by recent trade, and livestock movement [13], however here we present evidence that the introduction of this pathogen can be traced to much more ancient human migrations. We believe this to be an example of an opportunistic human pathogen reflecting ancient human dispersal patterns.

The recent and dramatic increase in the ability for extensive genomic sampling through whole genome sequencing coupled with extensive strain collections should enhance our ability to reconstruct even ancient epidemiological events. The strictly clonal reproductive patterns and low polymorphism frequency of evolutionarily stable molecular markers in *B. anthracis* makes this a model organism for tracking ancient epidemiological patterns. Whole genome sequencing of multiple *B. anthracis* strains has led to the construction of a highly accurate phylogenetic backbone [14] based upon an expansive world-wide strain collection [13]. Whereas it would be advantageous to sequence all available isolates within a lineage, this approach is still prohibitively expensive and requires SNP detection by whole genome comparisons. Targeting characters and taxa within specific lineages can further enhance the detection of evolutionary patterns which, when combined with sample spatial data, enables precise epidemiological tracking of disease, even for pre-historical events.

*B. anthracis* has dispersed globally via large and sequential radiations associated with human commerce and trade of animal products contaminated with *B. anthracis* spores [13]. Without human involvement, infected animals typically die within 7–10 days, seeding only the surrounding soil with spores thus keeping the spread of the disease relatively contained [15]. The potential for dispersion even among migratory herds is limited since infected animals typically die quickly before extensive dispersal can occur. Historically, an animal that died of anthrax was scavenged by people for its hair, hide, bones, and even consumed as food, facilitating the dispersal of spores away from a carcass. Indeed, imported spore-contaminated animal hides account for many of the recent US human anthrax cases [16], though such modern cases infrequently result in subsequent ecological establishment or further dispersal. Therefore, with the exception of the most recent human cases, the current distribution of *B. anthracis* can be traced to historical human dispersion, trade, and migratory patterns.

The most dramatic dispersal and clonal expansion of *B. anthracis* was the A-radiation [13], which is phylogenetically rooted in the Old World. Nested within the A-radiation is the highly successful trans-Eurasian subpopulation (TEA). Its prevalence in Europe and Asia is thought to be mediated by the east-west human trade routes, such as the “Silk Road”. One sublineage of this TEA population, western North America (WNA), was introduced into North America and has become highly successful within this geographic region. The WNA sublineage is dominant today in central Canada and much of the western United States.

In North America, two distinct types of anthrax cases are seen. Many have been observed along the East Coast and are associated with trade and industrial processing of contaminated animal products, often wool in textile mills [17]–[19]. These cases contribute to the overall genetic diversity of North American *B. anthracis* isolates, but generally represent small case clusters that do not become ecologically founded. This lack of establishment could be due to a requirement of suitable habitat for natural disease cycling [20]. In contrast, western North American grasslands are ideal for the ecological establishment of anthrax and may have persisted for much of the Holocene epoch, possibly over 10,000 years [21].

Indeed, at least two *B. anthracis* clades are ecologically established in North America on a sub-continental scale [13]. The “Ames” clade (A.Br.Ames) has been associated with highly localized and sporadic outbreaks in south Texas since at least the early 1980s [22]. Only a short evolutionary time period, very few SNPs, separate Texas Ames isolates from Asian near relatives, suggesting a recent or perhaps colonial animal importation. In contrast, the WNA clade has been widely successful both in distribution and frequency across central and northerly North American regions and is clearly ecologically established in many geographic areas. WNA isolates have been recovered from near the Artic Circle in Canada to the U.S. Mexican border and even in insular Haiti, and account for 89% of non-human anthrax cases in North America. The WNA clade also exhibits greater genetic diversity than the Ames clade, and a longer evolutionary separation (106 SNPs) from its nearest Old World relatives (TEA), suggesting a more ancient introduction into North America. The ecological dominance and disease importance of the WNA clade led us to examine its evolutionary history in greater detail using whole genome sequence analysis and highly accurate phylogenetic reconstructions.

## Results

### SNP Analyses

**Figure 1:**
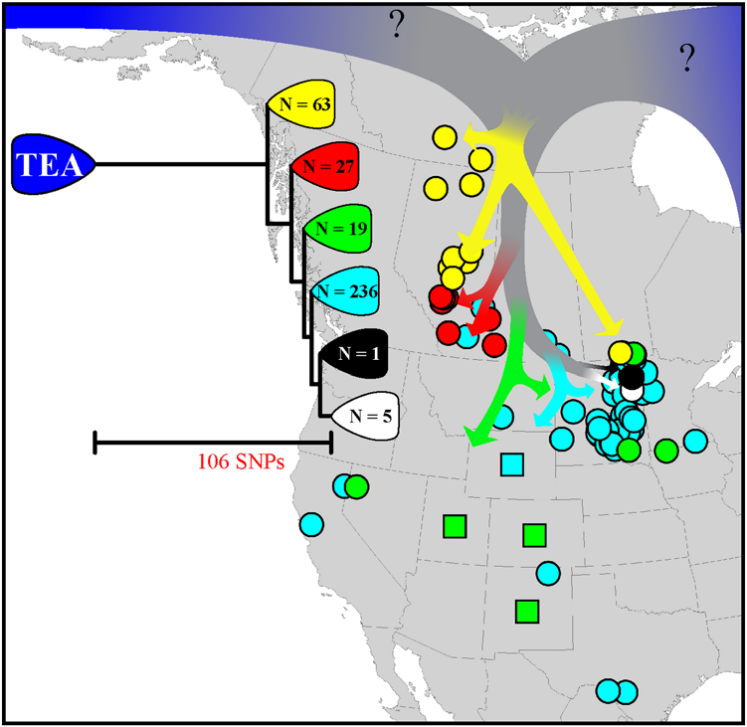
Phylogeography of the WNA *Bacillus anthracis* clone. The evolutionary tree of the dominant North American *B. anthracis* lineage (WNA) and mapping of isolate locations supports southern dispersal of anthrax in North America after founding by a European or Asian ancestor (TEA). Circles indicate precise GIS coordinates, squares indicate state-level information, and colors indicate phylogenetic grouping.

Whole genome sequencing of seven diverse strains led to the discovery of 2,850 SNPs suitable for conversion to whole genome tiling microarrays. One of these seven strains was the WNA strain (A0193) [14]. These SNPs were screened among 128 diverse isolates (described by MLVA15 analyses) and identified WNA as a monophyletic group rooted in the Old World TEA group. These data also showed the WNA group to be separated by a long phylogenetic branch (106 SNPs) (Fig. 1) representing 53% of the total distance from the initiation of the A branch radiation to the sequenced WNA isolate. Ten of these 106 SNPs were developed into Real-time PCR assays and used to screen all 387 isolates in the study. These SNPs identified six sub-clades within the previously described WNA lineage.

### Phylogenetic analyses

SNP characters are rare in the *B. anthracis* genome and almost never have character state reversals (∼0.1% homoplasy) [14]. Using a cladistic evolutionary model, there was one character-state inconsistency in these data (<1% homoplasy). Given such robust data, all approaches (e.g., MP, NJ) to construct phylogenetic trees generate the same evolutionary hypothesis (Fig. 1). Including any *B. anthracis* strain from outside this lineage allows us to accurately root this lineage using standard outgroup rooting methods.

### Geographical mapping of WNA isolates

The six sub-clades identified by SNP analyses revealed a north-south phylogeographic pattern (Fig. 1), with short terminal branches indicating rapid radiation of the WNA group following the initial establishment in northern Canada. Additionally, there were correlations among the nodes identified with recovery from their respective hosts. For example, the yellow group was mostly associated with wildlife (n = 63) whereas the other groups were almost exclusively from livestock.

## Discussion

SNPs are stable phylogenetic characters in *B. anthracis* and impart a highly accurate evolutionary hypothesis both in terms of branch lengths and branching order [14]. Our phylogenetic topologies are highly suggestive of an initial introduction of *B. anthracis* into the far north of North America and subsequent southerly dispersal (Fig. 1). The ancestral WNA phylogenetic nodes are northernmost, followed by progressively more southern locations for the more recently derived clades. More recent populations in southern states and a single human isolate from Haiti (not shown) are probably the result of recent commodity trading or infected livestock transport. In contrast, the northern-most and more ancient populations of WNA *B. anthracis* remain relatively localized, possibly due to association with bison (*Bison bison*) and restricted human commerce in this region. SNP-estimated evolutionary branch lengths provide additional support for a pre-Columbian introduction of WNA anthrax to North America. The accumulation of up to 106 SNPs since the split of the WNA lineage from the TEA lineage is a relatively large number for *B. anthracis* clades [14]. Interestingly, this indicates a long evolutionary separation followed by a genetic bottleneck and a single founding event in North America. In contrast, the Texas ecologically-established Ames lineage accumulated only ∼8 SNPs since introduction from Asia [23]. Molecular clock calibration is always problematic, but this >10-fold difference offers dramatic contrast between a recent and ancient New World introduction. Moreover, divergence times within branches of the A-radiation may be greatly affected by the number of generations in natural disease cycling. The model introduced in Van Ert et al (2007) was based upon 1 and 0.5 generations per year (approximately 3500 to 7000 ybp respectively for the major A-radiation). However, a recent overview of early outbreaks in Northern Canada provides greater insights into natural disease cycling in this region. Dragon and Elkin noted [24] that there were eight sporadic outbreaks between 1962 and 1991, and since 1993, there have been five more. Using these empirical data we can estimate 0.28 generations per year (13 outbreaks in 46 years). This empirical based estimation of this parameter significantly increases divergence times within the TEA-WNA lineage and suggests that divergence times provided in Van Ert et al. (2007) were underestimates.

Human migrations are the most likely source for the introduction and establishment of the WNA lineage of anthrax in North America through an ice-free corridor that connected Beringia to the southern areas east of the Rocky Mountains. Contiguous grassland habitat created by the partial retreat of the Laurentide and Cordilleran ice sheets would have been ideal for anthrax susceptible grazing herd animals. During the late Pleistocene epoch, Asia and North America were joined by the Beringian Steppe ecosystem [25]. This grassland refugium allowed animals and humans to freely cross what would become a water barrier (Bering Sea) in the Holocene. Humans could have transported *B. anthracis* to this land bridge from Asia and then moved it further south as the ice-free corridor and developing grassland opened in central Canada ∼13,000 ybp. Bison and other potential herding hosts were already widespread in North America, yet the limited range of the most ancient northern WNA *B. anthracis* populations suggest that anthrax was not present at this time south of the bottleneck created by the coalescing glaciers. Furthermore, as the ice-free corridor expanded, there was a simultaneous northward movement of these grazers like bison, yet the phylogenetic directionality of anthrax spread is southerly. Humans, however, did move southward through this corridor and could have brought contaminated animal products with them, which eventually enabled the ecological introduction of WNA *B. anthracis* in North America.

This northern-origin hypothesis is highly consistent with phylogeographic patterns and rates of SNP accumulation observed in recent *B. anthracis* isolates. While isolates from many different lineages have been observed in North America, their presence can be attributed to post colonial trade. Continent-wide dispersal of WNA *B. anthracis* may have involved later European colonizers, but some of the first inhabitants of the continent likely seeded the initial North American populations.

## Materials and Methods

### Strains

The 387 strains (Table 1) used in this study were obtained from various sources and linked to both livestock and wildlife outbreaks in North America. Four of these isolates were associated with human infections. Of the 387 strains, 352 cluster with the major ecologically established Western North American clade (WNA: A.Br.WNA) whereas 17 isolates were included to represent the TEA population as an outgroup. Genotypic data for each strain is provided in Table 2.

**Table 1:** Strains used in this study (Epidemiologic data)^1^.

*Sample ID* 2	*Country of Origin*	*Host*	*State/Province of Infection* 3	*County*	*Nearest City/Town*	*Latitude* 4	*Longitude*
A1055	USA	soil	LA				
A1065	USA	bovine	WY				
A2051	USA	Bovine	CA	Santa Clara	San Jose		
A2052	USA	Bovine	CA	Santa Clara	San Jose		
A0394	USA	goat kid	TX		Terrell		
A1115	USA	Bovine	TX	Leakey			
A1117	USA	Bovine	TX				
A2012	USA	Human	FL	Palm Bay			
A0169	Canada	bovine					
A0517	USA	Vaccine					
A0248	USA	Human					
A1057	USA	bovine	OK				
A1056	USA	Bovine					
A0078	USA						
A0156	USA	sheep					
A1058	USA	bovine	TX				
A1062	USA		MS				
A1063	USA		MD		Ft. Detrick		
A0032	China	Fur					
A0033	China	Wool					
A0149	Turkey	human					
A0241	Turkey	goat					
A0245	Turkey	bovine					
A0264	Turkey	human					
A0280	Italy	sheep					
A0303	Canada	bovine					
A0324	Slovakia						
A0343	Hungary						
A0362	Norway	bovine					
A0417	Hungary						
A0463	Pakistan	Sheep					
A0480	Russia	vaccine					
A0596	China	Marmot	Xinjiang				
A0604	China	Soil	Xinjiang				
A0610	China	Soil	Xinjiang				
A0669	China	Soil	Xinjiang				
2000031001	Canada						
2000031006	USA		NM				
2000031036	USA	Moose	WY				
2000031764		Goat					
2000031765							
2000031766	USA	Puritan Loom	NC				
2000031767							
2000031770							
2000031774							
2000032818							
2000032819							
2000032820							
2000032821							
2000032822							
2000032826							
2000032828							
2000032829							
2000032831							
2000032832							
2000032833							
2000032834							
2000032888							
2000032989							
2000032990							
2000032993							
2000032994							
2000032995							
2000032996							
2000032997							
2002013003							
2002013004							
2002013007							
2002013008							
2002013009							
2002013010							
2002013011							
2002013012							
2002013013							
2002013014							
2002013015							
2002013016							
2002013017							
2002013019							
2002013020							
2002013021							
2002013022							
2002013023							
2002013024							
2002013025							
2002013026							
2002013027							
2002013028							
2002013029							
2002013030							
2002013031							
2002013032							
2002013033							
2002013034							
2002013035							
2002013053							
2002013054							
2002013055							
2002013056							
2002013057							
2002013058							
2002013059							
2002013063							
2002013064							
2002013065							
2002013068							
2002013070	Canada	Bison					
2002013083	USA	Mice	WY				
2002013086							
2002013098							
2002721542	USA	Bovine	UT				
2002734033	USA		UT				
A0055	USA						
A0056	USA						
A0057	USA						
A0071	USA						
A0072	USA						
A0073	USA						
A0074	USA						
A0075	USA						
A0076	USA						
A0117	USA	Bovine	NV				
A0130	USA						
A0142	Canada	Moose					
A0143	Canada	bovine					
A0144	Canada	Bison					
A0157	USA	Pig					
A0162	Canada	Bison					
A0163	Canada	bovine					
A0165	Canada	Bison					
A0166	Canada	Bison					
A0167	Canada	bovine					
A0168	Canada	bovine					
A0170	Canada	bovine					
A0171	Canada	bovine					
A0172	Canada	bovine					
A0173	Canada	bovine					
A0174	Canada	bovine					
A0175	Canada	Bison					
A0176	Canada	Bison					
A0177	Canada	Bison					
A0178	Canada	Bison					
A0179	Canada	Bison					
A0180	Canada	bovine					
A0181	Canada	Ravens					
A0182	Canada	bovine					
A0192	Canada	bovine					
A0193	USA	bovine					
A0194	Canada	bovine					
A0195	Canada	bovine					
A0197	Canada	bovine					
A0198	Canada	bovine					
A0246	USA	bovine					
A0268	USA	Dog					
A0295	Canada	Bison					
A0296	Canada	Bison					
A0297	Canada	Bison					
A0300	Canada	bovine					
A0301	Canada	bovine					
A0302	Canada	bovine					
A0304	Canada	Bison					
A0305	Canada	Bison					
A0306	Canada	Bison					
A0307	Canada	bovine					
A0308	Canada	bovine					
A0309	Canada	bovine					
A0310	Canada	bovine					
A0311	Canada	bovine					
A0312	Canada	Bison					
A0313	Canada	Bison					
A0369	Canada	Bison					
A0374	USA	Bison					
A0377	Haiti	man					
A0383	USA	man	CO				
A0392	USA	bovine					
A0393	USA	bovine					
A0395	USA	deer	TX		Uvalde		
A0396	USA	bovine	TX		Del Rio		
A0397	USA	deer	TX		Del Rio		
A0398	USA	deer	TX		Del Rio		
A0407	USA	bovine					
A0408	USA	bovine					
A0409	USA	bovine					
A0410	USA	bovine					
A0418	Canada	Bison					
A0444	Canada	bovine					
A0445	Canada	bovine					
A0486	USA	bovine					
A0520	USA	WT deer					
A0526	USA	Bovine	MT		Billings		
A0767	Canada	Red Fox Scat	WBNP		Parson's Lake	59 45′ 53.9″ N	112 16′ 55.4″W
A0768	Canada	Red Fox Scat	WBNP		Parson's Lake	59 45′ 53.9″ N	112 16′ 55.4″W
A0770	Canada	Sand	WBNP		Parson's Lake	59 45′ 14.4″N	112 16′ 24.2″ W
A0771	Canada	Sand	WBNP		Parson's Lake	59 45′ 14.4″N	112 16′ 42.5″ W
A0772	Canada	Soil	WBNP		Falaise Lake	61 28′ 52.0″ N	116 15′ 54.0″ W
A0773	Canada	Soil	WBNP		Falaise Lake	61 28′ 52.0″ N	116 15′ 54.0″ W
A0774	Canada	Soil	WBNP		Falaise Lake	61 28′ 52.0″ N	116 15′ 54.0″ W
A0775	Canada	Soil	WBNP		Falaise Lake	61 28′ 52.0″ N	116 15′ 54.0″ W
A0776	Canada	Soil	WBNP		Falaise Lake	61 28′ 52.0″ N	116 15′ 54.0″ W
A0777	Canada	Soil	WBNP		Falaise Lake	61 28′ 52.0″ N	116 15′ 54.0″ W
A0778	Canada	Soil	WBNP		Falaise Lake	61 28′ 52.0″ N	116 15′ 54.0″ W
A0779	Canada	Soil	WBNP		Falaise Lake	61 28′ 52.0″ N	116 15′ 54.0″ W
A0780	Canada	Soil	WBNP		Falaise Lake	61 28′ 52.0″ N	116 15′ 54.0″ W
A0782	Canada	Soil	WBNP		Falaise Lake	61 29′ 7.0″ N	116 13′ 29.0″ W
A0783	Canada	Soil	WBNP		Falaise Lake	61 29′ 7.0″ N	116 13′ 29.0″ W
A0784	Canada	Soil	WBNP		Falaise Lake	61 29′ 7.0″ N	116 13′ 29.0″ W
A0785	Canada	Soil	WBNP		Falaise Lake	61 29′ 7.0″ N	116 13′ 29.0″ W
A0786	Canada	Soil	WBNP		Falaise Lake	61 29′ 7.0″ N	116 13′ 29.0″ W
A0788	Canada	Soil	WBNP		Falaise Lake	61 27′ 36.0″ N	116 18′ 23.0″ W
A0791	Canada						
A0792	Canada	Bovine			Rocky Mountain House		
A0793	Canada	Bovine			Rocky Mountain House		
A0794	Canada	Bovine			Rocky Mountain House		
A0795	Canada	Bovine			Rocky Mountain House		
A0796	Canada	Bovine			Rocky Mountain House		
A0797	Canada	Horse			Rocky Mountain House		
A0798	Canada	Bovine			Eckville		
A0799	Canada	Bovine			Eckville		
A0800	Canada	Bovine			Eckville		
A0801	Canada	Bovine			Eckville		
A0802	Canada	Bovine			Eckville		
A0803	Canada	Bovine			Rocky Mountain House		
A0804	Canada	Bovine			Caroline		
A0805	Canada	Bovine			Rocky Mountain House		
A0806	Canada	Bovine			Rocky Mountain House		
A0807	Canada	Bovine			Alhambra		
A0808	Canada	Bovine			Alhambra		
A0809	Canada	Bovine			Alhambra		
A0810	Canada	Bovine			Alhambra		
A0812	Canada	Bovine			Rocky Mountain House		
A0913	USA	Bovine	NV				
A0917	USA						
A0948	USA	Bovine	ND	Walsh	Park River		
A0949	USA	Equine	ND	Steele	Sharon		
A0950	USA	Bison	ND	Grand Forks	Grand Forks		
A0951	USA	Equine	ND	Traill	Portland		
A0952	USA	Bovine	MN	Clay	Hawley		
A0953	USA	Bovine	ND	Stutsman	Parkhurst		
A0954	USA	Bovine	ND	Foster	Grace City		
A0955	USA	Bovine	ND	Griggs	Cooperstown		
A0956	USA	Bovine	ND	Steele	Sharon		
A0957	USA						
A0958	USA	Bovine	ND	Nelson	Aneta		
A0959	USA	Bovine	ND	Grand Forks	Northwood		
A0960	USA	Bovine	ND	Traill	Portland		
A0961	USA	Equine	ND	Steele	Sharon		
A0962	USA	Bovine	ND	Grand Forks	Emerado		
A0963	USA	Bovine	MN	Becker	Lake Park		
A0964	USA	Bovine	ND	Grand Forks	Grand Forks		
A0965	USA	Bovine	ND	Pembina	Walhalla		
A0966	USA	Bovine	ND	Grand Forks	Emerado		
A0967	USA	Bovine	ND	Pembina	Hensel		
A0968	USA	Bovine	MN	Roseau	Greenbush		
A0969	USA	Bovine	ND	Grand Forks	Larimore		
A0970	USA	Bovine	ND	Adams	Reeder		
A0971	USA	Bovine	ND	Nelson	Aneta		
A0972	USA	Bovine	ND	Nelson	Pekin		
A0973	USA	Bovine	ND	Eddy	Hamar		
A0974	USA	Bovine	ND	Nelson	Tolna		
A0975	USA	Equine	ND	Nelson	Petersburg		
A0976	USA						
A0977	USA	Bovine	ND	Grand Forks	Larimore		
A0978	USA	Bovine	MN	Roseau	Badger		
A0979	USA	Bovine	MN	Mower	Lyle		
A0980	USA	Bovine	MN	Pennington	Thief River Falls		
A0981	USA	Bovine	ND	Grand Forks	Larimore		
A0982	USA	Bovine	ND	Pembina	Hensel		
A0983	USA						
A0984	USA						
A0985	USA						
A0988	Canada	Bison	WBNP				
A0989	Canada						
A0990	Canada						
A0991	Canada	Bear					
A0993	Canada	Bison					
A0994	Canada	Bison					
A0995	Canada	Wolf					
A0996	Canada	Bovine					
A0997	Canada	Bovine					
A0998	Canada	Moose					
A0999	Canada	Bovine					
A1000	Canada	Bovine					
A1001	Canada	Bovine	Manitoba		Vita		
A1002	Canada	Bovine	Manitoba		Vita		
A1003	Canada	Bovine	Manitoba		Vita		
A1004	Canada	Bovine	Manitoba		Vita		
A1005	Canada	Bovine	Manitoba		Vita		
A1006	Canada	Bovine	Manitoba		Vita		
A1007	Canada	Bovine	Manitoba		Vita		
A1008	Canada	Bovine	Manitoba		Vita		
A1009	Canada	Bovine	Manitoba		Vita		
A1010	Canada	Bovine	Manitoba		Vita		
A1011	Canada	Bovine	Manitoba		Vita		
A1012	Canada	Bovine	Manitoba		Vita		
A1013	Canada	Bovine	Manitoba		Vita		
A1014	Canada	Bovine	Manitoba		Vita		
A1015	Canada	Bovine	Manitoba		Vita		
A1016	Canada	Bovine	Manitoba		Vita		
A1017	Canada	Bovine	Manitoba		Vita		
A1018	Canada	Bovine	Manitoba		Vita		
A1019	Canada		Manitoba		Vita		
A1020	Canada		Manitoba		Vita		
A1021	Canada		Manitoba		Vita		
A1022	Canada	Bison	Manitoba		Vita		
A1023	Canada	Bovine	Manitoba		Vita		
A1024	Canada	Bovine	Manitoba		Vita		
A1025	Canada	Bovine	Manitoba		Vita		
A1026	Canada	Bovine	Manitoba		Vita		
A1027	Canada	Bovine	Manitoba		Vita		
A1028	Canada	Bovine	Manitoba		Vita		
A1029	USA	Bovine	NV		Vita		
A1030	USA	Bovine	NV	Washoe	Reno		
A1031	USA	Bovine	NV	Washoe	Reno		
A1040	USA	bovine	SD				
A1041	USA	bovine	SD				
A1042	USA	bovine	SD				
A1047	USA	bovine	IA				
A1118	USA	Bison	MN		Greenbush		
A1119	USA	Bovine	MN		Greenbush		
A1120	USA	Bovine	MN		Greenbush		
A1121	USA	Bovine	MN		Greenbush		
A1122	USA	Bovine	MN		Greenbush		
A1123	USA	Bovine	MN		Greenbush		
A1124	USA	Bovine	MN		Greenbush		
A1125	USA	Bovine	MN		Greenbush		
A1126	USA	Bovine	MN		Greenbush		
A1127	USA	Bovine	MN		Greenbush		
A1128	USA	Bovine	MN		Greenbush		
A1129	USA	Bovine	MN		Greenbush		
A1130	USA	Bovine	MN		Greenbush		
A1131	USA	Bovine	MN		Greenbush		
A1132	USA	Bovine	MN		Greenbush		
A1133	USA	Bovine	MN		Greenbush		
A1134	USA	Bovine	MN		Greenbush		
A1135	Canada		Manitoba		Sprague		
A1137	USA		SD				
A1138	USA		SD				
A1139	USA		SD				
A2014	Mexico	Bovine	Acuna				
A2015	Mexico	Bovine	Acuna				
A3455	USA	Bovine	SD			44.83663	100.33589
A3456	USA	Bovine	SD			45.66154	98.41541
A3457	USA	Bovine	SD			46.90731	98.10566
A3458	USA	Bovine	SD			44.77967	100.42004
A3459	USA	Bovine	SD			44.83672	100.33584
A3460	USA	Bovine	SD			44.70675	100.19478
A3461	USA	Bovine	SD			North	
A3462	USA	Bovine	SD			North	
A3463	USA	Bovine	SD			44.98544	100.36005
A3464	USA	Bovine	SD			44.69881	100.46175
A3465	USA	Bovine	SD			44.98094	100.30310
A3466	USA	Bovine	SD			44.68186	98.05983
A3467	USA	Bovine	SD			Central	
A3468	USA	Bovine	SD			44.38436	99.61700
A3469	USA	Bovine	SD			44.41030	99.80588
A3470	USA	Bovine	SD			44.72853	100.45644
A3471	USA	Bovine	SD			44.91887	99.79668
A3472	USA	Bovine	SD			44.72853	100.45644
A3473	USA	Bovine	SD			Central	
A3474	USA	Bovine	SD			44.67489	98.05903
A3475	USA	Bovine	SD			44.90751	100.48686
A3476	USA	Bovine	SD			North	
A3477	USA	Bovine	SD			Central	
A3478	USA	Bovine	SD			44.95312	100.49400
A3479	USA	Bovine	SD			45.70536	98.39379
A3480	USA	Bovine	SD			45.25863	100.23270
A3481	USA	Bovine	SD			44.58392	99.87045
A3482	USA	Bovine	SD			45.92200	98.05015
A3483	USA	Bovine	SD			North	
A3484	USA	Bovine	SD			44.94745	100.51275
A3485	USA	Bovine	SD			45.03621	100.30700
A3486	USA	Bovine	SD			45.16650	100.11835
A3487	USA	Bovine	SD			44.79834	100.55977
A3488	USA	Bovine	SD			44.95903	99.90616
A3489	USA	Bovine	SD			45.13020	100.33318
A3490	USA	Bovine	SD			44.84012	98.87670
A3491	USA	Bovine	SD			45.52951	97.72276
A3492	USA	Bovine	SD			45.35902	97.49963
A3493	USA	Bovine	SD			44.53633	103.51009
A3494	USA	Bovine	SD			44.91887	99.79668
A3495	USA	Bovine	SD			44.10121	99.86757
A3496	USA	Bovine	SD			Central	
A3497	USA	Bovine	SD			45.51513	100.48982
A3498	USA	Bovine	SD			43.78920	99.26104
A3499	USA	Bovine	SD			44.17555	99.50194
A3500	USA	Bovine	SD			44.10859	99.45870
A3501	USA	Bovine	SD			45.06766	100.39238

1 Missing or Unknown data has been intentionally left blank.

2 Samples having an “A” preceeding a four digit code are samples for which we possess live culture material. Samples having a 10-digit code were received as DNA from part of a historic collection maintained at the Centers for Disease Control and Prevention.

3 Two letter state codes are used when referencing states within the United States, otherwise the state is written out. WBNP is Wood Buffalo National Park.

4 Centroids were used for locations where state of origin information was available but Global Positioning System data was unavailable.

**Table 2:** Strains used in this study (Genotyping data)^1^.

Sample ID^2^	canSNP group^3^	A.Br.WNA^4^	wna237471	wna1141774	wna2994131	wna3368524	wna3631093	wna3682247	wna3732539	wna3774186	wna4461234	wna4718500
A1055	C.Br.A1055	A	T	C	C	A	G	G	T	T	G	G
A1065	C.Br.A1055	A	T	C	C	A	G	G	T	T	G	G
A2051	B.Br.001/002	A	T	C	C	A	G	G	T	T	G	G
A2052	B.Br.001/002	A	T	C	C	A	G	G	T	T	G	G
A0394	A.Br.001/002	A	T	C	C	A	G	G	T	T	G	G
A1115	A.Br.001/002	A	T	C	C	A	G	G	T	T	G	G
A1117	A.Br.001/002	A	T	C	C	A	G	G	T	T	G	G
A2012	A.Br.Ames	A	T	C	C	A	G	G	T	T	G	G
A0169	A.Br.001/002	A	T	C	C	A	G	G	T	T	G	G
A0517	A.Br.001/002	A	T	C	C	A	G	G	T	T	G	G
A0248	A.Br.Aust94	A	T	C	C	A	G	G	T	T	G	G
A1057	A.Br.Aust94	A	T	C	C	A	G	G	T	T	G	G
A1056	A.Br.003/004	A	T	C	C	A	G	G	T	T	G	G
A0078	A.Br.Vollum	A	T	C	C	A	G	G	T	T	G	G
A0156	A.Br.Vollum	A	T	C	C	A	G	G	T	T	G	G
A1058	A.Br.Vollum	A	T	C	C	A	G	G	T	T	G	G
A1062	A.Br.Vollum	A	T	C	C	A	G	G	T	T	G	G
A1063	A.Br.Vollum	A	T	C	C	A	G	G	T	T	G	G
A0032	A.Br.008/009	A	T	C	C	A	G	G	T	T	G	G
A0033	A.Br.008/009	A	T	C	C	A	G	G	T	T	G	G
A0149	A.Br.008/009	A	T	C	C	A	G	G	T	T	G	G
A0241	A.Br.008/009	A	T	C	C	A	G	G	T	T	G	G
A0245	A.Br.008/009	A	T	C	C	A	G	G	T	T	G	G
A0264	A.Br.008/009	A	T	C	C	A	G	G	T	T	G	G
A0280	A.Br.008/009	A	T	C	C	A	G	G	T	T	G	G
A0324	A.Br.008/009	A	T	C	C	A	G	G	T	T	G	G
A0343	A.Br.008/009	A	T	C	C	A	G	G	T	T	G	G
A0362	A.Br.008/009	A	T	C	C	A	G	G	T	T	G	G
A0417	A.Br.008/009	A	T	C	C	A	G	G	T	T	G	G
A0463	A.Br.008/009	A	T	C	C	A	G	G	T	T	G	G
A0480	A.Br.008/009	A	T	C	C	A	G	G	T	T	G	G
A0596	A.Br.008/009	A	T	C	C	A	G	G	T	T	G	G
A0604	A.Br.008/009	A	T	C	C	A	G	G	T	T	G	G
A0610	A.Br.008/009	A	T	C	C	A	G	G	T	T	G	G
A0669	A.Br.008/009	A	T	C	C	A	G	G	T	T	G	G
2000031001	A.Br.WNA	G	G	T	C	C	G	G	T	T	G	G
2000031006	A.Br.WNA	G	G	T	C	C	A	G	T	C	G	A
2000031036	A.Br.WNA	G	G	T	G	C	A	G	T	C	G	A
2000031764	A.Br.WNA	G	G	T	G	C	A	G	T	C	G	A
2000031765	A.Br.WNA	G	G	T	G	C	A	G	T	C	G	A
2000031766	A.Br.WNA	G	G	T	G	C	A	G	T	C	G	A
2000031767	A.Br.WNA	G	G	T	G	C	A	G	T	C	G	A
2000031770	A.Br.WNA	G	G	T	G	C	A	G	T	C	G	A
2000031774	A.Br.WNA	G	G	T	G	C	A	G	T	C	G	A
2000032818	A.Br.WNA	G	G	T	G	C	A	G	T	C	G	A
2000032819	A.Br.WNA	G	G	T	G	C	A	G	T	C	G	A
2000032820	A.Br.WNA	G	G	T	G	C	A	G	T	C	G	?
2000032821	A.Br.WNA	G	G	T	G	C	A	G	T	C	G	A
2000032822	A.Br.WNA	G	G	T	G	C	A	G	T	C	G	A
2000032826	A.Br.WNA	G	G	T	G	C	A	G	T	C	G	A
2000032828	A.Br.WNA	G	G	T	G	C	A	G	T	C	G	A
2000032829	A.Br.WNA	G	G	T	G	C	A	G	T	C	G	A
2000032831	A.Br.WNA	G	G	T	G	C	A	G	T	C	G	A
2000032832	A.Br.WNA	G	G	T	G	C	A	G	T	C	G	A
2000032833	A.Br.WNA	G	G	T	C	C	A	G	T	C	G	A
2000032834	A.Br.WNA	G	G	T	G	C	A	G	T	C	G	A
2000032888	A.Br.WNA	G	G	T	G	C	A	G	T	C	G	A
2000032989	A.Br.WNA	G	T	C	C	A	G	G	T	T	G	G
2000032990	A.Br.WNA	G	G	T	G	C	A	G	T	C	G	A
2000032993	A.Br.WNA	G	G	T	G	C	A	G	T	C	G	A
2000032994	A.Br.WNA	G	G	T	G	C	A	G	T	C	G	A
2000032995	A.Br.WNA	G	G	T	G	C	A	G	T	C	G	A
2000032996	A.Br.WNA	G	G	T	G	C	A	G	T	C	G	A
2000032997	A.Br.WNA	G	G	T	G	C	A	G	T	C	G	A
2002013003	A.Br.WNA	G	G	T	G	C	A	G	T	C	G	A
2002013004	A.Br.WNA	G	G	T	G	C	A	G	T	C	G	A
2002013007	A.Br.WNA	G	G	T	G	C	A	G	T	C	G	A
2002013008	A.Br.WNA	G	G	T	G	C	A	G	T	C	G	A
2002013009	A.Br.WNA	G	G	T	G	C	A	G	T	C	G	A
2002013010	A.Br.WNA	G	G	T	G	C	A	G	T	C	G	A
2002013011	A.Br.WNA	G	G	T	G	C	A	G	T	C	G	A
2002013012	A.Br.WNA	G	G	T	G	C	A	G	T	C	G	A
2002013013	A.Br.WNA	G	G	T	G	C	A	G	T	C	G	A
2002013014	A.Br.WNA	G	G	T	G	C	A	G	T	C	G	A
2002013015	A.Br.WNA	G	G	T	G	C	A	G	T	C	G	A
2002013016	A.Br.WNA	G	G	T	G	C	A	G	T	C	G	A
2002013017	A.Br.WNA	G	G	T	G	C	A	G	T	C	G	A
2002013019	A.Br.WNA	G	G	T	G	C	A	G	T	C	G	A
2002013020	A.Br.WNA	G	G	T	G	C	A	G	T	C	G	A
2002013021	A.Br.WNA	G	G	T	G	C	A	G	T	C	G	A
2002013022	A.Br.WNA	G	G	T	G	C	A	G	T	C	G	A
2002013023	A.Br.WNA	G	G	T	G	C	A	G	T	C	G	A
2002013024	A.Br.WNA	G	G	T	G	C	A	G	T	C	G	A
2002013025	A.Br.WNA	G	G	T	G	C	A	G	T	C	G	A
2002013026	A.Br.WNA	G	G	T	G	C	A	G	T	C	G	A
2002013027	A.Br.WNA	G	G	T	G	C	A	G	T	C	G	A
2002013028	A.Br.WNA	G	G	T	G	C	A	G	T	C	G	A
2002013029	A.Br.WNA	G	G	T	G	C	A	G	T	C	G	A
2002013030	A.Br.WNA	G	G	T	G	C	A	G	T	C	G	A
2002013031	A.Br.WNA	G	G	T	G	C	A	G	T	C	G	A
2002013032	A.Br.WNA	G	G	T	G	C	A	G	T	C	G	A
2002013033	A.Br.WNA	G	G	T	G	C	A	G	T	C	G	A
2002013034	A.Br.WNA	G	G	T	G	C	A	G	T	C	G	A
2002013035	A.Br.WNA	G	G	T	G	C	A	G	T	C	G	A
2002013053	A.Br.WNA	G	G	T	G	C	A	G	T	C	G	A
2002013054	A.Br.WNA	G	G	T	G	C	A	G	T	C	G	A
2002013055	A.Br.WNA	G	G	T	G	C	A	G	T	C	G	A
2002013056	A.Br.WNA	G	G	T	G	C	A	G	T	C	G	A
2002013057	A.Br.WNA	G	G	T	G	C	A	G	T	C	G	A
2002013058	A.Br.WNA	G	G	T	G	C	A	G	T	C	G	A
2002013059	A.Br.WNA	G	G	T	G	C	A	G	T	C	G	A
2002013063	A.Br.WNA	G	G	T	G	C	A	G	T	C	G	A
2002013064	A.Br.WNA	G	G	T	G	C	A	G	T	C	G	A
2002013065	A.Br.WNA	G	G	T	G	C	A	G	T	C	G	A
2002013068	A.Br.WNA	G	G	T	G	C	A	G	T	C	G	A
2002013070	A.Br.WNA	G	G	T	C	C	G	G	T	T	G	G
2002013083	A.Br.WNA	G	G	T	G	C	A	G	T	C	G	A
2002013086	A.Br.WNA	G	G	T	G	C	A	G	T	C	G	A
2002013098	A.Br.WNA	G	G	T	G	C	A	G	T	C	G	A
2002721542	A.Br.WNA	G	G	T	C	C	A	G	T	C	G	A
2002734033	A.Br.WNA	G	G	T	C	C	A	G	T	C	G	A
A0055	A.Br.WNA	G	G	T	G	C	A	G	T	C	G	A
A0056	A.Br.WNA	G	G	T	G	C	A	G	T	C	G	A
A0057	A.Br.WNA	G	G	T	G	C	A	G	T	C	G	A
A0071	A.Br.WNA	G	G	T	G	C	A	A	C	C	A	A
A0072	A.Br.WNA	G	G	T	G	C	A	A	T	C	A	A
A0073	A.Br.WNA	G	G	T	G	C	A	A	C	C	A	A
A0074	A.Br.WNA	G	G	T	G	C	A	A	C	C	A	A
A0075	A.Br.WNA	G	G	T	G	C	A	A	C	C	A	A
A0076	A.Br.WNA	G	G	T	G	C	A	G	T	C	G	A
A0117	A.Br.WNA	G	G	T	C	C	A	G	T	C	G	A
A0130	A.Br.WNA	G	G	T	G	C	A	A	C	C	A	A
A0142	A.Br.WNA	G	G	T	C	C	G	G	T	T	G	G
A0143	A.Br.WNA	G	G	T	C	C	A	G	T	T	G	A
A0144	A.Br.WNA	G	G	T	C	C	G	G	T	T	G	G
A0157	A.Br.WNA	G	G	T	G	C	A	G	T	C	G	A
A0162	A.Br.WNA	G	G	T	C	C	G	G	T	T	G	G
A0163	A.Br.WNA	G	G	T	G	C	A	G	T	C	G	A
A0165	A.Br.WNA	G	G	T	C	C	G	G	T	T	G	G
A0166	A.Br.WNA	G	G	T	C	C	G	G	T	T	G	G
A0167	A.Br.WNA	G	G	T	G	C	A	G	T	C	G	A
A0168	A.Br.WNA	G	G	T	G	C	A	G	T	C	G	A
A0170	A.Br.WNA	G	G	T	G	C	A	G	T	C	G	A
A0171	A.Br.WNA	G	G	T	G	C	A	G	T	C	G	A
A0172	A.Br.WNA	G	G	T	G	C	A	G	T	C	G	A
A0173	A.Br.WNA	G	G	T	C	C	A	G	T	T	G	A
A0174	A.Br.WNA	G	G	T	C	C	A	G	T	T	G	A
A0175	A.Br.WNA	G	G	T	C	C	G	G	T	T	G	G
A0176	A.Br.WNA	G	G	T	C	C	G	G	T	T	G	G
A0177	A.Br.WNA	G	G	T	C	C	G	G	T	T	G	G
A0178	A.Br.WNA	G	G	T	C	C	G	G	T	T	G	G
A0179	A.Br.WNA	G	G	T	C	C	G	G	T	T	G	G
A0180	A.Br.WNA	G	G	T	C	C	A	G	T	T	G	A
A0181	A.Br.WNA	G	G	T	C	C	G	G	T	T	G	G
A0182	A.Br.WNA	G	G	T	C	C	G	G	T	T	G	G
A0192	A.Br.WNA	G	G	T	G	C	A	G	T	C	G	A
A0193	A.Br.WNA	G	G	T	C	C	A	G	T	C	G	A
A0194	A.Br.WNA	G	G	T	C	C	A	G	T	T	G	A
A0195	A.Br.WNA	G	G	T	C	C	G	G	T	T	G	G
A0197	A.Br.WNA	G	G	T	C	C	G	G	T	T	G	G
A0198	A.Br.WNA	G	G	T	C	C	G	G	T	T	G	G
A0246	A.Br.WNA	G	G	T	C	C	A	G	T	C	G	A
A0268	A.Br.WNA	G	G	T	G	C	A	G	T	C	G	A
A0295	A.Br.WNA	G	G	T	C	C	G	G	T	T	G	G
A0296	A.Br.WNA	G	G	T	C	C	G	G	T	T	G	G
A0297	A.Br.WNA	G	G	T	C	C	G	G	T	T	G	G
A0300	A.Br.WNA	G	G	T	C	C	G	G	T	T	G	G
A0301	A.Br.WNA	G	G	T	C	C	A	G	T	T	G	A
A0302	A.Br.WNA	G	G	T	C	C	A	G	T	T	G	A
A0303	A.Br.WNA	A^5^	G	T	C	C	G	G	T	T	G	G
A0304	A.Br.WNA	G	G	T	C	C	G	G	T	T	G	G
A0305	A.Br.WNA	G	G	T	C	C	G	G	T	T	G	G
A0306	A.Br.WNA	G	G	T	C	C	G	G	T	T	G	G
A0307	A.Br.WNA	G	G	T	C	C	G	G	T	T	G	G
A0308	A.Br.WNA	G	G	T	C	C	G	G	T	T	G	G
A0309	A.Br.WNA	G	G	T	C	C	G	G	T	T	G	G
A0310	A.Br.WNA	G	G	T	C	C	G	G	T	T	G	G
A0311	A.Br.WNA	G	G	T	C	C	G	G	T	T	G	G
A0312	A.Br.WNA	G	G	T	C	C	G	G	T	T	G	G
A0313	A.Br.WNA	G	G	T	C	C	G	G	T	T	G	G
A0369	A.Br.WNA	G	G	T	C	C	G	G	T	T	G	G
A0374	A.Br.WNA	G	G	T	C	C	A	G	T	C	G	A
A0377	A.Br.WNA	G	G	T	G	C	A	G	T	C	G	A
A0383	A.Br.WNA	G	G	T	C	C	A	G	T	C	G	A
A0392	A.Br.WNA	G	G	T	G	C	A	G	T	C	G	A
A0393	A.Br.WNA	G	G	T	G	C	A	G	T	C	G	A
A0395	A.Br.WNA	G	G	T	G	C	A	G	T	C	G	A
A0396	A.Br.WNA	G	G	T	G	C	A	G	T	C	G	A
A0397	A.Br.WNA	G	G	T	G	C	A	G	T	C	G	A
A0398	A.Br.WNA	G	G	T	G	C	A	G	T	C	G	A
A0407	A.Br.WNA	G	G	T	G	C	A	G	T	C	G	A
A0408	A.Br.WNA	G	G	T	G	C	A	G	T	C	G	A
A0409	A.Br.WNA	G	G	T	G	C	A	G	T	C	G	A
A0410	A.Br.WNA	G	G	T	G	C	A	G	T	C	G	A
A0418	A.Br.WNA	G	G	T	C	C	G	G	T	T	G	G
A0444	A.Br.WNA	G	G	T	G	C	A	G	T	C	G	A
A0445	A.Br.WNA	G	G	T	G	C	A	G	T	C	G	A
A0486	A.Br.WNA	G	G	T	G	C	A	G	T	C	G	A
A0520	A.Br.WNA	G	G	T	G	C	A	G	T	C	G	A
A0526	A.Br.WNA	G	G	T	G	C	A	G	T	C	G	A
A0767	A.Br.WNA	G	G	T	C	C	G	G	T	T	G	G
A0768	A.Br.WNA	G	G	T	C	C	G	G	T	T	G	G
A0770	A.Br.WNA	G	G	T	C	C	G	G	T	T	G	G
A0771	A.Br.WNA	G	G	T	C	C	G	G	T	T	G	G
A0772	A.Br.WNA	G	G	T	C	C	G	G	T	T	G	G
A0773	A.Br.WNA	G	G	T	C	C	G	G	T	T	G	G
A0774	A.Br.WNA	G	G	T	C	C	G	G	T	T	G	G
A0775	A.Br.WNA	G	G	T	C	C	G	G	T	T	G	G
A0776	A.Br.WNA	G	G	T	C	C	G	G	T	T	G	G
A0777	A.Br.WNA	G	G	T	C	C	G	G	T	T	G	G
A0778	A.Br.WNA	G	G	T	C	C	G	G	T	T	G	G
A0779	A.Br.WNA	G	G	T	C	C	G	G	T	T	G	G
A0780	A.Br.WNA	G	G	T	C	C	G	G	T	T	G	G
A0782	A.Br.WNA	G	G	T	C	C	G	G	T	T	G	G
A0783	A.Br.WNA	G	G	T	C	C	G	G	T	T	G	G
A0784	A.Br.WNA	G	G	T	C	C	G	G	T	T	G	G
A0785	A.Br.WNA	G	G	T	C	C	G	G	T	T	G	G
A0786	A.Br.WNA	G	G	T	C	C	G	G	T	T	G	G
A0788	A.Br.WNA	G	G	T	C	C	G	G	T	T	G	G
A0791	A.Br.WNA	G	G	T	C	C	G	G	T	T	G	G
A0792	A.Br.WNA	G	G	T	C	C	A	G	T	T	G	A
A0793	A.Br.WNA	G	G	T	C	C	A	G	T	T	G	A
A0794	A.Br.WNA	G	G	T	C	C	A	G	T	T	G	A
A0795	A.Br.WNA	G	G	T	C	C	A	G	T	T	G	A
A0796	A.Br.WNA	G	G	T	C	C	A	G	T	T	G	A
A0797	A.Br.WNA	G	G	T	C	C	A	G	T	T	G	A
A0798	A.Br.WNA	G	G	T	C	C	A	G	T	T	G	A
A0799	A.Br.WNA	G	G	T	C	C	A	G	T	T	G	A
A0800	A.Br.WNA	G	G	T	C	C	A	G	T	T	G	A
A0801	A.Br.WNA	G	G	T	C	C	A	G	T	T	G	A
A0802	A.Br.WNA	G	G	T	C	C	A	G	T	T	G	A
A0803	A.Br.WNA	G	G	T	C	C	A	G	T	T	G	A
A0804	A.Br.WNA	G	G	T	C	C	A	G	T	T	G	A
A0805	A.Br.WNA	G	G	T	C	C	A	G	T	T	G	A
A0806	A.Br.WNA	G	G	T	C	C	A	G	T	T	G	A
A0807	A.Br.WNA	G	G	T	C	C	A	G	T	T	G	A
A0808	A.Br.WNA	G	G	T	C	C	A	G	T	T	G	A
A0809	A.Br.WNA	G	G	T	C	C	A	G	T	T	G	A
A0810	A.Br.WNA	G	G	T	C	C	A	G	T	T	G	A
A0812	A.Br.WNA	G	G	T	C	C	A	G	T	T	G	A
A0913	A.Br.WNA	G	G	T	C	C	A	G	T	C	G	A
A0917	A.Br.WNA	G	G	T	C	C	A	G	T	C	G	A
A0948	A.Br.WNA	G	G	T	G	C	A	G	T	C	G	A
A0949	A.Br.WNA	G	G	T	G	C	A	G	T	C	G	A
A0950	A.Br.WNA	G	G	T	G	C	A	G	T	C	G	A
A0951	A.Br.WNA	G	G	T	G	C	A	G	T	C	G	A
A0952	A.Br.WNA	G	G	T	G	C	A	G	T	C	G	A
A0953	A.Br.WNA	G	G	T	G	C	A	G	T	C	G	A
A0954	A.Br.WNA	G	G	T	G	C	A	G	T	C	G	A
A0955	A.Br.WNA	G	G	T	G	C	A	G	T	C	G	A
A0956	A.Br.WNA	G	G	T	G	C	A	G	T	C	G	A
A0957	A.Br.WNA	G	G	T	G	C	A	G	T	C	G	A
A0958	A.Br.WNA	G	G	T	G	C	A	G	T	C	G	A
A0959	A.Br.WNA	G	G	T	G	C	A	G	T	C	G	A
A0960	A.Br.WNA	G	G	T	G	C	A	G	T	C	G	A
A0961	A.Br.WNA	G	G	T	G	C	A	G	T	C	G	A
A0962	A.Br.WNA	G	G	T	G	C	A	G	T	C	G	A
A0963	A.Br.WNA	G	G	T	G	C	A	G	T	C	G	A
A0964	A.Br.WNA	G	G	T	G	C	A	G	T	C	G	A
A0965	A.Br.WNA	G	G	T	G	C	A	G	T	C	G	A
A0966	A.Br.WNA	G	G	T	G	C	A	G	T	C	G	A
A0967	A.Br.WNA	G	G	T	G	C	A	G	T	C	G	A
A0968	A.Br.WNA	G	G	T	G	C	A	G	T	C	G	A
A0969	A.Br.WNA	G	G	T	G	C	A	G	T	C	G	A
A0970	A.Br.WNA	G	G	T	G	C	A	G	T	C	G	A
A0971	A.Br.WNA	G	G	T	G	C	A	G	T	C	G	A
A0972	A.Br.WNA	G	G	T	G	C	A	G	T	C	G	A
A0973	A.Br.WNA	G	G	T	G	C	A	G	T	C	G	A
A0974	A.Br.WNA	G	G	T	G	C	A	G	T	C	G	A
A0975	A.Br.WNA	G	G	T	G	C	A	G	T	C	G	A
A0976	A.Br.WNA	G	G	T	G	C	A	G	T	C	G	A
A0977	A.Br.WNA	G	G	T	G	C	A	G	T	C	G	A
A0978	A.Br.WNA	G	G	T	G	C	A	G	T	C	G	A
A0979	A.Br.WNA	G	G	T	G	C	A	G	T	C	G	A
A0980	A.Br.WNA	G	G	T	G	C	A	G	T	C	G	A
A0981	A.Br.WNA	G	G	T	G	C	A	G	T	C	G	A
A0982	A.Br.WNA	G	G	T	G	C	A	G	T	C	G	A
A0983	A.Br.WNA	G	G	T	G	C	A	G	T	C	G	A
A0984	A.Br.WNA	G	G	T	G	C	A	G	T	C	G	A
A0985	A.Br.WNA	G	G	T	G	C	A	G	T	C	G	A
A0988	A.Br.WNA	G	G	T	C	C	G	G	T	T	G	G
A0989	A.Br.WNA	G	G	T	C	C	G	G	T	T	G	G
A0990	A.Br.WNA	G	G	T	C	C	G	G	T	T	G	G
A0991	A.Br.WNA	G	G	T	C	C	G	G	T	T	G	G
A0993	A.Br.WNA	G	G	T	C	C	G	G	T	T	G	G
A0994	A.Br.WNA	G	G	T	C	C	G	G	T	T	G	G
A0995	A.Br.WNA	G	G	T	C	C	G	G	T	T	G	G
A0996	A.Br.WNA	G	G	T	G	C	A	G	T	C	G	A
A0997	A.Br.WNA	G	G	T	G	C	A	G	T	C	G	A
A0998	A.Br.WNA	G	G	T	C	C	G	G	T	T	G	G
A0999	A.Br.WNA	G	G	T	G	C	A	G	T	C	G	A
A1000	A.Br.WNA	G	G	T	G	C	A	G	T	C	G	A
A1001	A.Br.WNA	G	G	T	G	C	A	G	T	C	G	A
A1002	A.Br.WNA	G	G	T	G	C	A	G	T	C	G	A
A1003	A.Br.WNA	G	G	T	G	C	A	G	T	C	G	A
A1004	A.Br.WNA	G	G	T	G	C	A	G	T	C	G	A
A1005	A.Br.WNA	G	G	T	G	C	A	G	T	C	G	A
A1006	A.Br.WNA	G	G	T	G	C	A	G	T	C	G	A
A1007	A.Br.WNA	G	G	T	G	C	A	G	T	C	G	A
A1008	A.Br.WNA	G	G	T	G	C	A	G	T	C	G	A
A1009	A.Br.WNA	G	G	T	G	C	A	G	T	C	G	A
A1010	A.Br.WNA	G	G	T	G	C	A	G	T	C	G	A
A1011	A.Br.WNA	G	G	T	G	C	A	G	T	C	G	A
A1012	A.Br.WNA	G	G	T	G	C	A	G	T	C	G	A
A1013	A.Br.WNA	G	G	T	G	C	A	G	T	C	G	A
A1014	A.Br.WNA	G	G	T	G	C	A	G	T	C	G	A
A1015	A.Br.WNA	G	G	T	G	C	A	G	T	C	G	A
A1016	A.Br.WNA	G	G	T	G	C	A	G	T	C	G	A
A1017	A.Br.WNA	G	G	T	G	C	A	G	T	C	G	A
A1018	A.Br.WNA	G	G	T	G	C	A	G	T	C	G	A
A1019	A.Br.WNA	G	G	T	G	C	A	G	T	C	G	A
A1020	A.Br.WNA	G	G	T	G	C	A	G	T	C	G	A
A1021	A.Br.WNA	G	G	T	G	C	A	G	T	C	G	A
A1022	A.Br.WNA	G	G	T	C	C	G	G	T	T	G	G
A1023	A.Br.WNA	G	G	T	G	C	A	G	T	C	G	A
A1024	A.Br.WNA	G	G	T	G	C	A	G	T	C	G	A
A1025	A.Br.WNA	G	G	T	G	C	A	G	T	C	G	A
A1026	A.Br.WNA	G	G	T	G	C	A	G	T	C	G	A
A1027	A.Br.WNA	G	G	T	G	C	A	G	T	C	G	A
A1028	A.Br.WNA	G	G	T	C	C	A	G	T	C	G	A
A1029	A.Br.WNA	G	G	T	C	C	A	G	T	C	G	A
A1030	A.Br.WNA	G	G	T	G	C	A	G	T	C	G	A
A1031	A.Br.WNA	G	G	T	G	C	A	G	T	C	G	A
A1040	A.Br.WNA	G	G	T	C	C	A	G	T	C	G	A
A1041	A.Br.WNA	G	G	T	C	C	A	G	T	C	G	A
A1042	A.Br.WNA	G	G	T	C	C	A	G	T	C	G	A
A1047	A.Br.WNA	G	G	T	G	C	A	G	T	C	G	A
A1118	A.Br.WNA	G	G	T	G	C	A	G	T	C	G	A
A1119	A.Br.WNA	G	G	T	G	C	A	G	T	C	G	A
A1120	A.Br.WNA	G	G	T	G	C	A	G	T	C	G	A
A1121	A.Br.WNA	G	G	T	G	C	A	G	T	C	G	A
A1122	A.Br.WNA	G	G	T	G	C	A	G	T	C	G	A
A1123	A.Br.WNA	G	G	T	G	C	A	G	T	C	G	A
A1124	A.Br.WNA	G	G	T	G	C	A	G	T	C	G	A
A1125	A.Br.WNA	G	G	T	G	C	A	G	T	C	G	A
A1126	A.Br.WNA	G	G	T	G	C	A	G	T	C	G	A
A1127	A.Br.WNA	G	G	T	G	C	A	G	T	C	G	A
A1128	A.Br.WNA	G	G	T	G	C	A	G	T	C	G	A
A1129	A.Br.WNA	G	G	T	G	C	A	G	T	C	G	A
A1130	A.Br.WNA	G	G	T	G	C	A	G	T	C	G	A
A1131	A.Br.WNA	G	G	T	G	C	A	G	T	C	G	A
A1132	A.Br.WNA	G	G	T	G	C	A	G	T	C	G	A
A1133	A.Br.WNA	G	G	T	G	C	A	G	T	C	G	A
A1134	A.Br.WNA	G	G	T	G	C	A	G	T	C	G	A
A1135	A.Br.WNA	G	G	T	G	C	A	G	T	C	G	A
A1137	A.Br.WNA	G	G	T	C	C	A	G	T	C	G	A
A1138	A.Br.WNA	G	G	T	C	C	A	G	T	C	G	A
A1139	A.Br.WNA	G	G	T	C	C	A	G	T	C	G	A
A2014	A.Br.WNA	G	G	T	G	C	A	G	T	C	G	A
A2015	A.Br.WNA	G	G	T	G	C	A	G	T	C	G	A
A3455	A.Br.WNA	G	G	T	G	C	A	G	T	C	G	A
A3456	A.Br.WNA	G	G	T	G	C	A	G	T	C	G	A
A3457	A.Br.WNA	G	G	T	G	C	A	G	T	C	G	A
A3458	A.Br.WNA	G	G	T	G	C	A	G	T	C	G	A
A3459	A.Br.WNA	G	G	T	G	C	A	G	T	C	G	A
A3460	A.Br.WNA	G	G	T	G	C	A	G	T	C	G	A
A3461	A.Br.WNA	G	G	T	G	C	A	G	T	C	G	A
A3462	A.Br.WNA	G	G	T	G	C	A	G	T	C	G	A
A3463	A.Br.WNA	G	G	T	G	C	A	G	T	C	G	A
A3464	A.Br.WNA	G	G	T	G	C	A	G	T	C	G	A
A3465	A.Br.WNA	G	G	T	G	C	A	G	T	C	G	A
A3466	A.Br.WNA	G	G	T	G	C	A	G	T	C	G	A
A3467	A.Br.WNA	G	G	T	G	C	A	G	T	C	G	A
A3468	A.Br.WNA	G	G	T	G	C	A	G	T	C	G	A
A3469	A.Br.WNA	G	G	T	G	C	A	G	T	C	G	A
A3470	A.Br.WNA	G	G	T	G	C	A	G	T	C	G	A
A3471	A.Br.WNA	G	G	T	G	C	A	G	T	C	G	A
A3472	A.Br.WNA	G	G	T	G	C	A	G	T	C	G	A
A3473	A.Br.WNA	G	G	T	G	C	A	G	T	C	G	A
A3474	A.Br.WNA	G	G	T	G	C	A	G	T	C	G	A
A3475	A.Br.WNA	G	G	T	G	C	A	G	T	C	G	A
A3476	A.Br.WNA	G	G	T	G	C	A	G	T	C	G	A
A3477	A.Br.WNA	G	G	T	G	C	A	G	T	C	G	A
A3478	A.Br.WNA	G	G	T	G	C	A	G	T	C	G	A
A3479	A.Br.WNA	G	G	T	G	C	A	G	T	C	G	A
A3480	A.Br.WNA	G	G	T	G	C	A	G	T	C	G	A
A3481	A.Br.WNA	G	G	T	G	C	A	G	T	C	G	A
A3482	A.Br.WNA	G	G	T	G	C	A	G	T	C	G	A
A3483	A.Br.WNA	G	G	T	G	C	A	G	T	C	G	A
A3484	A.Br.WNA	G	G	T	G	C	A	G	T	C	G	A
A3485	A.Br.WNA	G	G	T	G	C	A	G	T	C	G	A
A3486	A.Br.WNA	G	G	T	G	C	A	G	T	C	G	A
A3487	A.Br.WNA	G	G	T	G	C	A	G	T	C	G	A
A3488	A.Br.WNA	G	G	T	G	C	A	G	T	C	G	A
A3489	A.Br.WNA	G	G	T	G	C	A	G	T	C	G	A
A3490	A.Br.WNA	G	G	T	G	C	A	G	T	C	G	A
A3491	A.Br.WNA	G	G	T	G	C	A	G	T	C	G	A
A3492	A.Br.WNA	G	G	T	G	C	A	G	T	C	G	A
A3493	A.Br.WNA	G	G	T	G	C	A	G	T	C	G	A
A3494	A.Br.WNA	G	G	T	G	C	A	G	T	C	G	A
A3495	A.Br.WNA	G	G	T	G	C	A	G	T	C	G	A
A3496	A.Br.WNA	G	G	T	G	C	A	G	T	C	G	A
A3497	A.Br.WNA	G	G	T	G	C	A	G	T	C	G	A
A3498	A.Br.WNA	G	G	T	G	C	A	G	T	C	G	A
A3499	A.Br.WNA	G	G	T	G	C	A	G	T	C	G	A
A3500	A.Br.WNA	G	G	T	G	C	A	G	T	C	G	A
A3501	A.Br.WNA	G	G	T	G	C	A	G	T	C	G	A

1 Genotyping data are presented as the SNP state at the particular locus. Each locus is presented with reference to the genome position in the Ancestral Ames strain (GenBank ID: AE017334).

2 Sample IDs with “A” numbers indicate isolates for which we have live culture. Sample IDs bearing a 10-digit number are from an historical strain collection maintained by the Center for Disease Control and Prevention.

3 Canonical SNP groups as defined in Van Ert et al. (2007).

4 The A.Br.WNA canonical SNP was also definied in Van Ert et al. (2007).

5 Rules of parsimony place this isolate (A0303) in the A.Br.WNA group. This single data point is homoplastic.

### DNA Extraction

DNA for Affymetrix whole genome tiling microarrays was extracted on 21 geographically dispersed and genetically diverse (MLVA15) strains within the A.Br.009 clade by a modification of the chloroform∶isoamyl alcohol method described in Keim et al. (1997). DNA for sub-clade specific TaqMan® MGB dual-probe real-time PCR SNP assays was extracted by a simplified heat lysis protocol described in Keim et al. (2000).

### SNP Discovery

Seven diverse *B. anthracis* strains, including one from the WNA clade, which had previously been characterized by Multi-Locus Variable Number of Tandem Repeats Analyses (MLVA), were further characterized by shotgun cloning and whole genome sequencing by the Sanger method. These efforts lead to the discovery of ∼3,000 SNPs within the *B. anthracis* genome.

### Construction of a whole genome tiling microarray

A custom Affymetrix genotyping microarray was constructed using 2850 SNPs. We genotyped 128 diverse strains using this format; relevant to this study, 10 strains were from the trans-Eurasian population (TEA: A.Br.008/009) and 21 were from the Western North American clade (WNA: A.Br.WNA). Of the 2,850 SNPs, 78 separated TEA from WNA, while 28 split the WNA clade into 6 genotypes (Fig. 1).

### Real-Time PCR analyses

We selected 10 SNPs for conversion into Taqman® MGB dual-probe real-time PCR SNP assays (Table 3). Assays were designed using Primer Express (Applied Biosystems, Foster City, CA). This is a highly sensitive technology that is fast and cost-effective when analyzing hundreds of samples. Table 4 describes the associated phylogenetic groups for each SNP: Branch separating TEA from WNA (4 SNPs) and WNA subtypes (6 SNPs). These rapid PCR assays were used to genotype a total of 352 isolates from the WNA clade (Table 2). Most of the WNA members had been previously identified using the canonical SNP assay for A.Br.009 [13].

**Table 3:** Primers and Probes used to detect WNA clade specific SNPs.

*Locus Name* 1 *(GenBank ID: AE017334)*	*Primers 5′→3′* 2	*Probes 5′→3′* 3
**wna2994131**	F-GCACGGTCTTTCTAAATTCATTGTT	VIC-AAAGAACATAGGAGTTTAC
	R-TGCGATTGGAGTTGCAAATAAT	FAM-AAAGAACATACGAGTTTAC
**wna3631093**	F-CAGAACCTACAGAACCATCATTAAAGAA	VIC-ATTGCTTACACTTCAGA
	R-GTAAACCCATTACCACCACACTATGT	FAM-ATTGCTTACACTTCGGAC
**wna3682247**	F-ACATGTTCACTTCACACATTTTCTCA	VIC-ACTCTTGAACAAACCA
	R-GCAATTGCAACAAGTCATCCA	FAM-ACTCTTGAACAAGCCAA
**wna3732539**	F-CTAAAAGCTCCAAATGCAATAGCA	VIC-CTGTTCCTGATAACAA
	R-TGGTGGATCAAATGCAGTTAACTT	FAM-CTGTTCCTGATAATAA
**wna3774186**	F-CTTTGGTTTTCCTTTGGTATAATCTCTT	VIC-ATGGCACCTTTACATCT
	R-TGACGTTGAAGGATGGAATATTTTTA	FAM-CAGATGGCATCTTT
**wna4461234**	F-TTTTGATGGAGAGATTTTGCTTTCT	VIC-TACGTTCTACAAATGGTACGT
	R-GCGAAATCGAGCAAGGATTC	FAM-TACGTTCTACAGATGGT
**wna4718500**	F-GCATCACCATTTAGATCATAAACCA	VIC-AGCCATGTGTATAGAA
	R-TGTCGTCGTACAGAAAGAAACGTT	FAM-AGCCATGTGTATGGAA
**wna237471**	F-TCGATGGTGCGAGCTTTTATATT	VIC-AATGAGCTCGGCACCAT
	R-TGGTCATTGGTGGTATTTGCA	FAM-AATGAGCTCTGCACCA
**wna1141774**	F-CGGCTTTTTTTCATTACGCATTA	VIC-CATTTACCGTATTGTTTTG
	R-AAAGAAAACAGAACATGCATTGATG	FAM-ATTTACCGCATTGTTTTG
**wna3368524**	F-CACGCTTATCGCCATCGAT	VIC-TCTACTGGCATTTCA
	R-TGACGGAAGTGTAACGGAAGGT	FAM-TCTACTGGAATTTC

1 The locus name correlates to the position on the whole genome sequence of the Ancestral Ames strain (AE017334).

2 F refers to the Forward primer whereas R refers to the Reverse primer.

3 Allele states are designated in red. VIC is the fluor conjugated to the 5′ end of the probe for one allele whereas FAM is the fluor conjugated to the 5′end of the alternate allele.

**Table 4:** SNPs used in this study to define clades in WNA.

*Branch Name* 1	*Locus Name*	*Base Change (Ancestral→Derived)*
A.Br.009^2^	canSNP2589947*	A→G
A.Br.018^2^	wna0237471	T→G
A.Br.018^2^	wna1141774	C→T
A.Br.018^2^	wna3368524	A→C
A.Br.019^3^	wna3631093	A→G
A.Br.019^3^	wna4718500	G→A
A.Br.020^4^	wna3774186	T→C
A.Br.021^5^	wna2994131	C→G
A.Br.022^6^	wna3682247	A→G
A.Br.022^6^	wna4461234	G→A
A.Br.023^7^	wna3732539	T→C

1 SNPs are arranged in this table from basal to derived nodes.

* The A.Br.009 canonical SNP was previously reported^5^ and was used to identify most of the WNA clade samples used in this study.

2 These four SNPs are located on the Fig. 1 basal node separating TEA from the “yellow” clade.

3 These two SNPs are located on the Fig. 1 node separating the “yellow” from “red” clades.

4 This SNP is located on the Fig. 1 node separating the “red” from “green” clades.

5 This SNP is located on the Fig. 1 node separating the “green” from “blue” clades.

6 These two SNPs are located on the Fig. 1 node separating the “blue” from “black” clades.

7 This SNP is located on the Fig. 1 node separating the “black” and the terminal clades.

### Spatial Analysis

Spatial data were then linked to genotypic analysis for each isolate and plotted with a Geographic Information System (ArcView 3.3). Some isolates were retrieved from archival collections and were not associated with geographic information, hence only 285 isolates were spatially mapped.

### Phylogenetic analysis

Phylogenetic analysis using a cladistic approach was accomplished with PAUP 4.0 [26].
