# Correction: Pre-Columbian Origins for North American Anthrax

**DOI:** 10.1371/annotation/9e8af820-8037-4f98-86b3-6581e16c2ae6

**Published:** 2009-05-20

**Authors:** Leo J. Kenefic, Talima Pearson, Richard T. Okinaka, James M. Schupp, David M. Wagner, Alex R. Hoffmaster, Carla B. Trim, Wai-Kwan Chung, Jodi A. Beaudry, Lingxia Jiang, Pawel Gajer, Jeffrey T. Foster, James I. Mead, Jacques Ravel, Paul Keim

**Affiliations:** 1 Department of Biological Sciences, Northern Arizona University, Flagstaff, Arizona, United States of America; 2 The Institute for Genome Sciences, University of Maryland School of Medicine, Baltimore, Maryland, United States of America; 3 Bacterial Zoonoses Branch, Centers for Disease Control and Prevention, Atlanta, Georgia, United States of America; 4 Pathogen Genomics Division, Translational Genomics Research Institute, Phoenix, Arizona, United States of America

There were several errors in the author byline.

Drs. Lingxia Jiang and Pawel Gajer were mistakenly omitted from the author byline. Lingxia Jiang should be listed as the tenth author and is affiliated with the Institute for Genome Sciences, University of Maryland School of Medicine, Baltimore, Maryland, United States of America. Her contributions were as follows: Optimized the Affymetrix tiling chip protocol and processed all the Affymetrix tiling GeneChips (>125). Contributed to the analysis of the GeneChip data.

Pawel Gajer should be listed as the 11th author and is affilated with the Institute for Genome Sciences, University of Maryland School of Medicine, Baltimore, Maryland, United States of America. His contributions were as follows: Developed the algorithm to extract SNP data from the microbial Affymetrix whole genome tiling GeneChip. Processed the GeneChip and extracted SNP data.

The eighth author's name appears incorrectly. It should be: Carla B. Trim.

The sixth author, Jacques Ravel, should appear as the fourteenth author.

